# Tislelizumab combined with apatinib in the treatment of advanced renal clear cell carcinoma: a case report

**DOI:** 10.1097/CAD.0000000000001241

**Published:** 2021-08-27

**Authors:** Fanjie Qu, Shuang Wu, Jie Zhang

**Affiliations:** Department of Oncology, Dalian Third People’s Hospital, Dalian, China

**Keywords:** apatinib, renal clear cell carcinoma, tislelizumab

## Abstract

Most patients with advanced renal cancer develop drug resistance to targeted drugs, and the disease progresses with the prolongation of the treatment cycle. Therefore, it is necessary to explore new treatment methods for advanced renal cancer to obtain continuous efficacy and prolong the survival time of patients. The patient was diagnosed with advanced renal cancer that had progressed after previous antiangiogenic drug therapy, based on the clinical course and imaging findings. The patient was treated with ‘tislelizumab plus apatinib’. The clinical discomfort symptoms were quickly relieved after treatment, and the evaluation two cycles later showed stable disease. After two cycles of continuation of the original regimen, reevaluation computed tomography demonstrated a significant reduction in the size of the abdominal cavity mass and the therapeutic evaluation was partial remission after four cycles; however, the patient developed abnormal liver function after treatment, manifested as nausea and poor appetite, and significantly increased bilirubin and transaminase levels, which were considered as immune-related liver injuries. After glucocorticoid treatment, the patient’s condition quickly improved and recovered. This report is the first to suggest a potential approach to advanced renal clear cell carcinoma and describes the effects of immunocombination therapy on advanced renal clear cell carcinoma; the results showed the current stage success of the immunocombination treatment, suggesting that this treatment may be an effective treatment option for patients with advanced renal clear cell carcinoma. In addition, the toxic and side effects of combined immunotherapy need to be carefully identified by every doctor. Since only one patient with advanced renal cancer was observed in this report, the clinical data are very limited and further observation and accumulation of more experience are needed, and further clinical studies will be conducted on the efficacy and safety of this combination regimen.

## Introduction

In the past 10 years, targeted therapy has greatly improved the prognosis of patients with advanced renal cell carcinoma (RCC) in China. The overall survival (OS) has increased from 13 months in the era of cytokines to about 30 months, which is a milestone in the treatment of advanced renal cancer [1,2]. However, most patients develop drug resistance to targeted drugs, and the disease progresses with prolonged treatment cycles [2]. Meanwhile, the benefit of high-risk groups was not obvious. Therefore, it is necessary to explore new treatment methods for advanced renal cancer to obtain continuous efficacy and prolong the survival time of patients. Immunotherapy represented by immune checkpoint inhibitors (ICIs) has achieved great success, not only changing the pattern of malignant tumor treatment, but also leading the direction of tumor therapy in recent years [3].

## Case report

A 56-year-old woman was admitted with a 3-month history of shortness of breath and low back pain in November 2017. PET/ computed tomography (CT) showed that there was a lesion at the upper pole of the left renal, accompanied by the spleen and adrenal involvement, with multiple subpleural hypermetabolic nodules and multiple retroperitoneal hypermetabolic foci in the right lung, which were considered as metastatic tumors with right pleural effusion; adenocarcinoma cells were found in the pleural effusion. Cellular immunohistochemistry confirmed renal clear-cell carcinoma. The patient was diagnosed with stage IV renal clear cell carcinoma and was administered first-line treatment with sorafenib from December 2017, and was administered 800 mg/day. Three months later, the right pleural effusion disappeared completely, and the lesion in the abdominal cavity was stable, and radical nephrectomy and splenectomy were performed on 19 June 2018. Postoperative pathology: (left renal) clear cell carcinoma, with multiple necrosis (approximately 50%) and inflammatory cell infiltration; nuclear classification: grade 3; tumor invasion of the renal capsule, tumor size: 11 × 7 × 7 cm, no cancer involvement in the adrenal gland, spleen or surrounding adipose tissue of the renal; targeted therapy was discontinued 6 weeks before and after surgery; sorafenib was continued after surgery for 2 months and was voluntarily discontinued in September 2018. One year after drug withdrawal, CT showed multiple masses in the operative area and adjacent to the left erector spinae muscle, and involvement in the adjacent intestinal tract, and postoperative recurrence was considered in September 2019.

As second-line therapy, pazopanib was prescribed for 8 months and the best therapeutic evaluation was stable disease, and pazopanib was administered at 800 mg/day. The lesion began to increase slowly half a year later, and the patient developed abdominal pain, accompanied by nausea and vomiting and weight loss in May 2020.

**Fig. 1:**
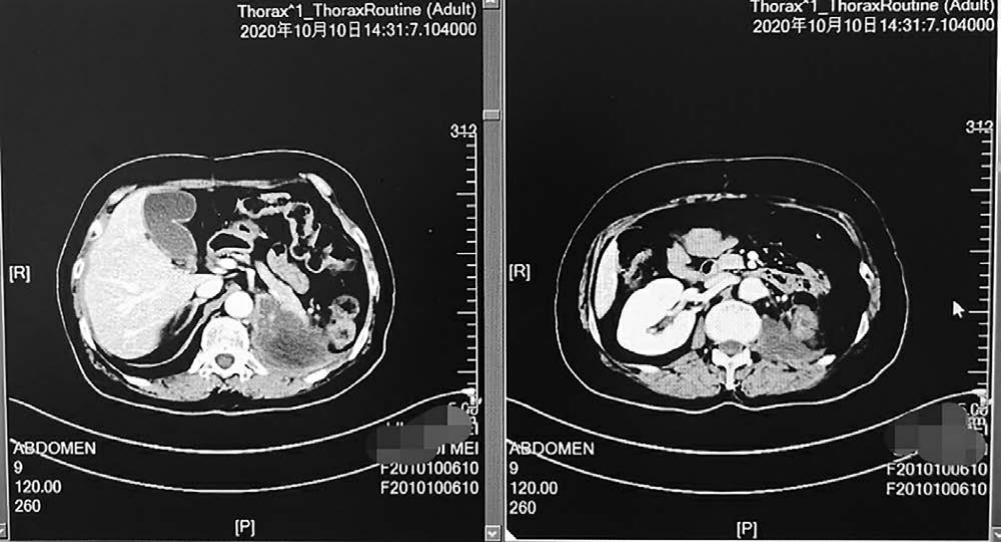
Computed tomography shows the lesions in the abdominal cavity before the regimen of ‘tislelizumab plus apatinib’ was given.

Reevaluation of CT demonstrated a significant increase in the size of the abdominal cavity mass, and the greater curvature of the stomach wall was more likely to be invaded. Therefore, everolimus was administered as a third-line treatment in May 2020, and everolimus was administered at 800 mg/day. Oxycodone hydrochloride was administered for pain relief and symptomatic treatment. Genetic testing was recommended, and during the treatment with everolimus, the abdominal pain was still aggravated, accompanied by nausea and vomiting. The weight loss was nearly 5 kg, and reevaluation imaging demonstrated that the lesions in the abdominal cavity were significantly enlarged (Fig. 1). The patient developed a poor general condition and had a performance staus of 2.

**Fig. 2:**
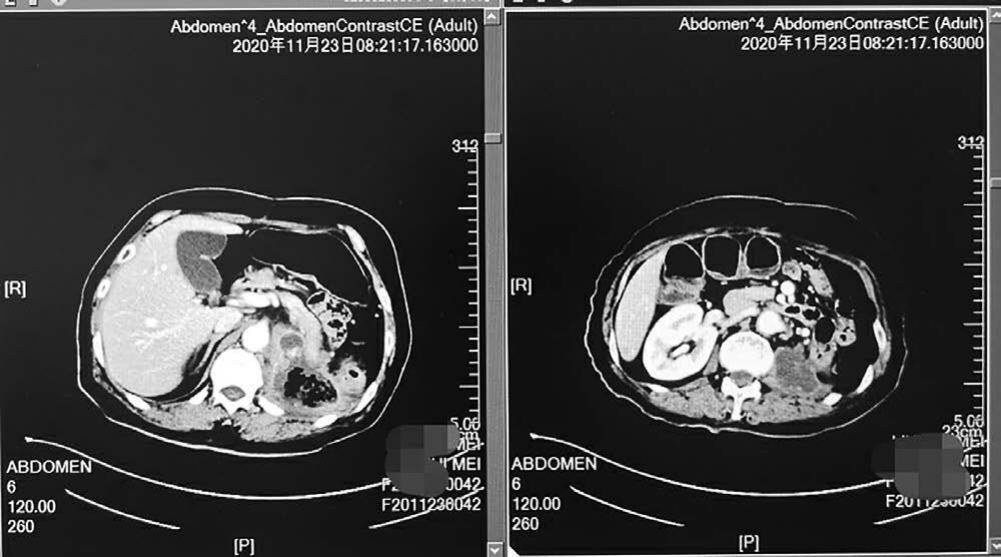
Computed tomography shows the lesions in the abdominal cavity after the regimen of ‘tislelizumab plus apatinib’ was given for two cycles.

The patient underwent genetic testing (Illumina sequencing platform) in September 2020. Gene detection showed that (plasma) tumor mutation burden (TMB) was 9.65 mutations/MB, and the position was 86.08% (TMB was classified into low, medium and high in accordance with the order of the subjects in this sample library; sort from lowest to highest, by position <25% is low, position ≥25 and <75% is medium and position ≥75% is high), so the patient was determined to have a high TMB. Therefore, the regimen of ‘tislelizumab plus apatinib’ was initiated as a four-line treatment from October 2020; tislelizumab was given 200 mg/time every 3 weeks and apatinib was given 250 mg/day. The symptoms of abdominal pain, nausea and poor appetite were significantly improved after 1 cycle. Reevaluation two cycles later (Fig. 2) showed stable disease, and the dosage of oxycodone hydrochloride was gradually reduced. After three cycles, abdominal pain was relieved, painkillers were discontinued, food intake was improved and weight gained and performance staus of the case improved from 2 to 0.

**Fig. 3:**
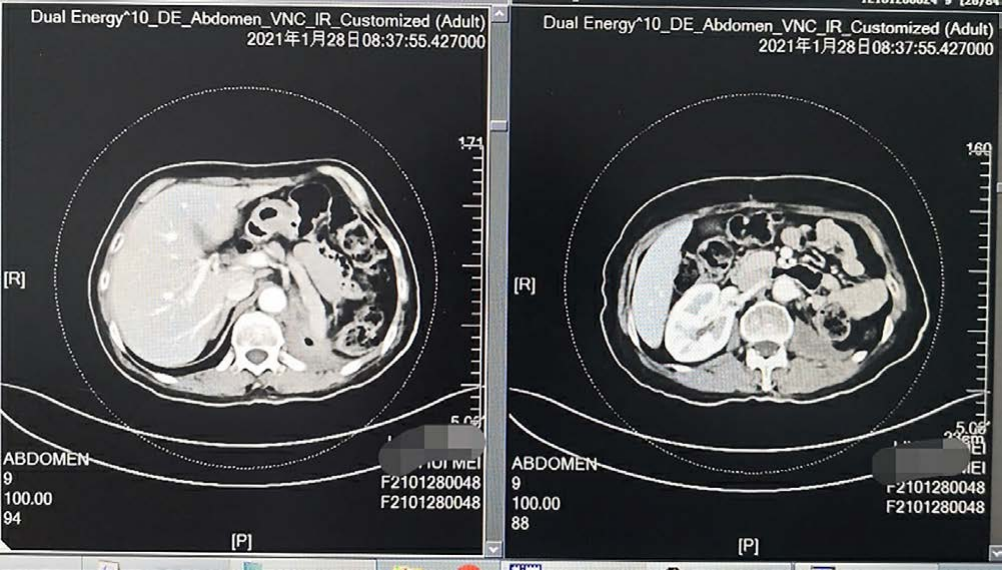
Computed tomography shows the lesions in the abdominal cavity after the regimen of ‘tislelizumab plus apatinib’ was given for four cycles.

Reevaluation of CT demonstrated significant reduction in the size of abdominal cavity mass (Fig. 3) and the therapeutic evaluation was partial remission after four cycles. But the patient developed nausea and poor appetite again. Liver function test (28 January 2021) included: serum total bilirubin 29.2  µmol/L↑, serum direct bilirubin 11.7 µmol/L↑, serum alanine aminotransferase 224 U/L↑, serum aspartate aminotransferase 147 U/L↑, serum γ-glutamyltransferase 1337 U/L↑ and serum lactate dehydrogenase 237 U/L. The patients were immediately administered an intravenous infusion of methylprednisolone 40 mg/day for immune-related hepatitis caused by ralicizumab, along with liver protectant drugs such as glutathione and magnesium isoglycyrrhizate, and the symptoms of nausea and poor appetite were significantly improved after 3 days of treatment with methylprednisolone. Reexamination of liver function test include (31 January 2021): serum total bilirubin 20.1 µmol/L, serum direct bilirubin 7.5 µmol/L↑, serum alanine aminotransferase 183 U/L↑, serum aspartate aminotransferase 70 U/L↑, serum γ-glutamyltransferase 1242 U/L↑ and lactate dehydrogenase 217 U/L. Reexamination of liver function test (3 February 2021) conducted after 3 days of continued treatment includes: serum total bilirubin 14.5 µmol/L, serum direct bilirubin 5.6 µmol/L↑, serum alanine aminotransferase 120 U/L↑, serum aspartic aminotransferase 36 U/L and serum γ-glutamyltransferase 1111 U/L↑. The intravenous infusion of methylprednisone was replaced with oral prednisone 35 mg/day after 6 days of treatment, and the dose was reduced by 5 mg every 5 days until it was reduced to 5 mg/day on 6 March 2021. During this period, repeated reexaminations showed that the liver function returned to normal and remained stable; however, the general condition of the patient was significantly improved, the weight of the patient increased by 10 kg and the toxicities experienced by the patient were mainly hand–foot skin reaction and hypertension, which were well managed.

**Fig. 4:**
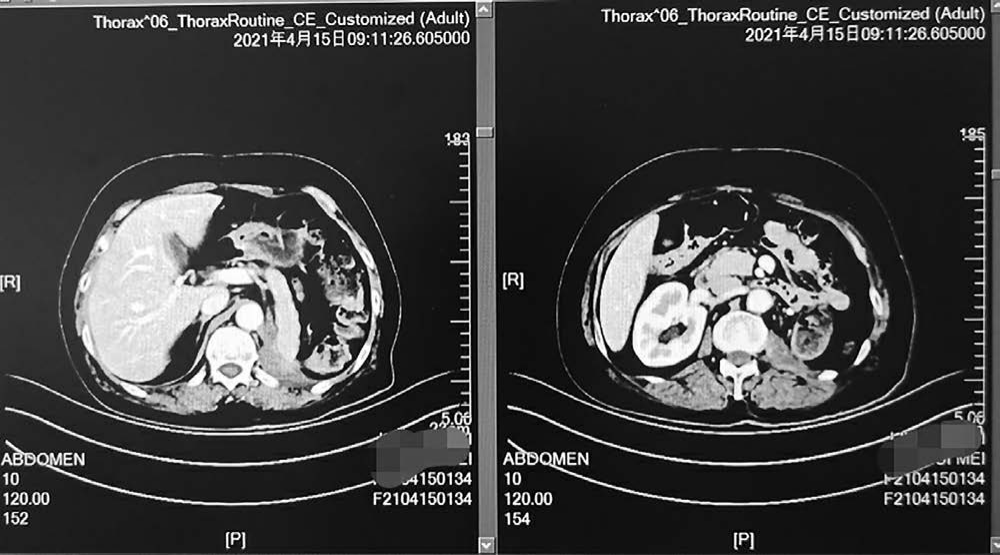
Computed tomography shows the lesions in the abdominal cavity after the regimen of ‘tislelizumab plus apatinib’ was given for 6 months.

The treatment of ‘tislelizumab + apatinib’ resumed on 10 March 2021. Liver function test results were normal on 1 April 2021, and at the same time, oral prednisone was discontinued. Reevaluation CT demonstrated a continual decrease in the size of abdominal cavity lesions on 15 April 2021(Fig. 4), and partial remission was maintained; at present, the patient achieved progression-free survival (PFS) of 7 months, and she also developed a general condition. She is still undergoing regular treatment and follow-up by June2021.

In this case report, informed consent was obtained before each treatment. The patients’ responses to all drugs were according to the Response Evaluation Criteria in Solid Tumors (RECIST) criteria, and adverse events were as per the Common Terminology Criteria for Adverse Events criteria.

## Discussion

Immunotherapy has shown some efficacy in patients with advanced metastatic RCC who have progressed during targeted drug therapy [4,5]. The objective response rate (ORR) of nivolumab monotherapy in patients with advanced/metastatic RCC with TKI resistance was 20–30%, with a median OS of 20–25 months [5]. Based on the results of the checkmate 025 trial, nivolumab was approved as a second-line treatment for metastatic RCC after failure of vascular endothelial growth factor (VEGF) targeting by the Food and Drug Administration (FDA) in November 2015. McDermott *et al*., [6] confirmed that atezolizumab (PD-L1 inhibitor) alone had an ORR of 15% and a median PFS of 5.6 months in patients with previously treated RCC. However, the results of these studies show that the effect of a single drug of PD-1/PD-L1 inhibitor in patients with advanced RCC is not more advantageous than that of targeted drugs, and the efficiency is low in practical applications. Therefore, combination therapy, especially combined antivascular therapy, has become the mainstream strategy of immunotherapy.

A review of current clinical data shows that immunotherapy combined with antiangiogenic therapy is effective for advanced RCC. Nazzani *et a*l., [7] first confirmed the efficacy of ICIs combined with antiangiogenic agents in the first-line or backline treatment of advanced RCC in some phase I studies. At least three phase III studies were successful in 2019. Immotion151 is the first phase III study with positive results in advanced RCC, which showed that atezolizumab combined with bevacizumab significantly prolonged PFS in PD-L1 positive patients compared to the sunitinib monotherapy group [8]. Subsequently, based on the positive results of the phase III Keynote-426 study [9] and the JAVELIN Renal 101 study [10], FDA approved pembrolizumab in combination with Axitinib and avelumab in combination with Axitinib for first-line treatment of advanced RCC in April and May 2019, respectively. Based on these findings, the 2020 National Comprehensive Cancer Network Renal Cancer Guidelines include a standard first-line recommendation that a combination of immune and antivascular targeted therapy can be used for advanced, medium-high risk renal clear cell carcinoma compared to the previous version of the 2019 Guidelines [11].

However, immunotherapy has started late, and the cost of imported immunotherapy is high in China. Considering the availability of drugs, the cost of immunocombination therapy, the complexity of adverse reactions caused by combination therapy, indications and medical insurance policies, targeted drugs are still the first choice for most patients with advanced RCC in China. Treatment failure is more common in first-line RCC patients who do not receive immunocombined antiangiogenic therapy [7–10].

Tislelizumab injection is a humanized IgG4 anti-PD-1 antibody drug with innovative structural modification independently developed by China and is the only PD-1 mAb that has been genetically engineered in the Fc segment of the antibody [12], avoiding antibody-dependent cell-mediated phagocytosis [13], which enables the drug to have stronger antitumor activity, and its Fab segment can specifically bind to PD-1, so its affinity is higher than that of similar antibodies and the probability of off-target effect is low [12,14]. The results of the BGB-A317-204 study showed that the median OS of patients with advanced urothelial carcinoma treated with Tislelizumab was 9.8 months, and the ORR was 24.8% [15]. The results of phase III clinical trial, RATIONALE 307, showed that Tislelizumab + paclitaxel/paclitaxel (albumin-bound) + carboplatin significantly prolonged the PFS of patients to 7.7 months compared with chemotherapy alone and compared with 5.5 months in the control group. The study also showed that immunocombination therapy has a high response rate, with more than 70% of patients achieving objective response, and the duration of curative effect is longer [16].

Apatinib, a selective inhibitor of vascular endothelial growth factor receptor-2 (VEGFR-2) tyrosine kinase, which blocks downstream signal transduction and has a strong antitumor angiogenesis effect, has been proven to be well tolerated and effective for the treatment of a broad range of advanced solid tumors [17]. As for the dosage of apatinib combined with PD-1 mAb, preclinical studies have shown that, based on a syngeneic lung cancer mouse model, low-dose apatinib significantly alleviates hypoxia and potently increases the infiltration of lymphocytes in tumors, and modestly suppresses tumor growth *in vivo*; high-dose apatinib is inferior to low-dose apatinib in hindering the recruitment of immunosuppressive myeloid cells; low-dose apatinib potently reduces transforming growth factor-β levels, and demonstrated that combining low-dose apatinib with anti-PD-L1 antibody significantly retarded tumor growth and metastases, and induced prolonged survival in mouse models, and provided the rationale for the combination of PD-1/PD-L1 blockade and low-dose VEGFR2-TKI in tumors [18].

This patient failed from the third-line treatment, with extensive metastasis in the abdominal cavity, relatively large tumor load, accompanied by the clinical symptoms of nausea, poor appetite, abdominal pain and emaciation *et al*.Based on the above study results, we selected Tislelizumab immunotherapy combined with a lower dose of apatinib (250 mg, once daily) after thorough communication with patients. The clinical discomfort symptoms were quickly relieved after treatment and the evaluation two cycles later showed stable disease according to RECIST. After two cycles of continuation of the original regimen, reevaluation CT demonstrated a significant reduction in the size of the abdominal cavity mass and the therapeutic evaluation was partial remission after four cycles; however, the patient developed abnormal liver function after four cycles of treatment, manifested as nausea and poor appetite, and significantly increased bilirubin and transaminase levels, which were considered as immune-related liver injuries. After glucocorticoid treatment, the patient quickly improved and recovered, and the dose of glucocorticoid was gradually reduced to 5 mg/day, and the treatment resumed after 1 month when the lesion continued to maintain the partial remission status.

In this case, we found that the blood TMB was high through genetic testing before treatment. Previous studies have shown that TMB can be used to predict the efficacy of immunotherapy, although it is not a perfect biomarker [19]. TMB has been reported to be highly correlated with the efficacy of PD-1/PD-L1 inhibitors in recent years, and high TMB, which represents genomic instability, is considered to have the potential to induce antigen production and further enhance immunogenicity [20]. Current studies have confirmed that TMB can be used as a clinical screening biomarker for the use of ICIs in melanoma, lung cancer and urothelial carcinoma [21–24]. However, a significant proportion of patients with advanced cancer do not have enough tumor tissue available for molecular testing; therefore, whether circulating tumor DNA (ctDNA), namely blood TMB (BTMB), can be used as a noninvasive method to predict the efficacy of immunotherapy has also attracted widespread attention [20].

In fact, there are many kinds of antivascular targeted therapies and ICIs in clinical practice, and the choice of immunocombination therapy in the treatment of advanced renal cancer will indeed bring confusion to clinicians. We applied Tislelizumab combined with low-dose apatinib in this patient, and achieved satisfactory results; however, when the patient is administered immunotherapy, it should be noted that attention must be paid to common adverse reactions such as rash, diarrhea and liver function injury; vigilance should also be raised for rare serious adverse reactions such as immune-associated pneumonia, immune-associated myocarditis and myasthenia gravis. It should also be noted that the onset time of the PD-1 mAb is longer than that of traditional drugs. In this case, the lesion did not shrink after the initial two cycles of treatment, and the efficacy reached partial remission after the 4th cycle of treatment. If the action characteristics of PD-1 mAb are not realized, the drug may be stopped prematurely, which may lead to the failure of subsequent treatment; therefore, we suggest that the efficacy of PD-1 should be evaluated after treatment was administered for at least 2–4 cycles. The fact that the patient’s lesions continued to remission also confirms that the onset time of this immunotherapy is different from that of traditional therapy. At present, the patient is still undergoing maintenance treatment with a PFS of 6 months, which exceeds the average survival time of previously reported cases.

In conclusion, the authors suggest that the prognosis of advanced clear cell renal carcinoma after multiline therapy is very poor. For patients with advanced clear cell renal carcinoma who have previously received targeted therapy, it is very important to explore a treatment method that is suitable for patients with posterior line therapy to extend the survival period as much as possible and improve the quality of life of patients.

In this study, tirelizumab combined with apatinib was used in the treatment of one patient with advanced RCC, and the current stage of success was achieved, suggesting that this treatment mode may be an effective treatment option for patients with advanced renal clear cell carcinoma. In addition, the toxic and side effects of combined immunotherapy need to be carefully identified by every doctor. Toxicity and side effects can be handled correctly only by making a clear judgment.

Because only one patient with advanced renal cancer was observed in this report, the clinical data are very limited and further observation and accumulation of more experience are needed, and further clinical studies will be conducted on the efficacy and safety of this combination regimen. We hope to encourage researchers to pay attention to patients with advanced posterior line treatment of renal cancer to improve the quality of life and survival rate of patients.

## Acknowledgements

This study was approved by the institutional review board of Dalian Third People’s Hospital. Written informed consent was obtained from all the patients. No funding was received for this study.

Q.F., Z.J. and W.S. were responsible for collecting data, sorting out data and writing the article; Q.F. was responsible for guiding the writing and participating in the revision of the article, and all authors read and approved the final manuscript.

## Conflicts of interest

There are no conflicts of interest.
